# Industrial Validation of a Promising Functional Strain of *Lactobacillus plantarum* to Improve the Quality of Italian Sausages

**DOI:** 10.3390/microorganisms8010116

**Published:** 2020-01-15

**Authors:** Daniela Campaniello, Barbara Speranza, Antonio Bevilacqua, Clelia Altieri, Maria Rosaria Corbo, Milena Sinigaglia

**Affiliations:** Department of the Science of Agriculture, Food and Environment (SAFE), University of Foggia, 71122 Foggia, Italy; daniela.campaniello@unifg.it (D.C.); barbara.speranza@unifg.it (B.S.); clelia.altieri@unifg.it (C.A.); milena.sinigaglia@unifg.it (M.S.)

**Keywords:** functional starter cultures, meat, validation, *Lactobacillus plantarum*

## Abstract

This paper proposes the industrial validation of a functional strain of *Lactobacillus plantarum* (strain 178). First, acidification in a meat model medium and bioactivity towards *Staphylococcus aureus*, *Salmonella* sp., *Listeria monocytogenes*, and *Escherichia coli* were assessed; the performances of *Lb. plantarum* 178 were compared to those of a commercial *Lb. sakei* and a probiotic *Lb. casei*. *Lb. plantarum* 178 inhibited the pathogens and experienced a higher acidification at 15 °C. *Lb. casei* and *Lb. plantarum* were used for an industrial fermentation of traditional Italian sausages. The strains assured the correct course of fermentation and inhibited pathogens and enterobacteria. This study represents the scaling up and the validation of a promising strain at industrial level and shows the possibility of performing the fermentation of traditional Italian sausage through functional starter cultures, combining the benefit of a controlled fermentation and possible health benefits.

## 1. Introduction

The awareness of consumers of the importance of some foods in diet is increasing, along with the knowledge of the benefits derived from the use of certain microorganisms—therefore, thanks to new biotechnologies, some traditional processes have been modified to improve the quality of the final product. In particular, fermentation processes can be significantly improved by modern technology and biotechnology [1]. Fermentations are driven by microbial starters added to raw materials—they assure food safety and shelf-life, reduce variability, and enhance organoleptic characteristics [1].

The fermentation of sausages involves the participation of mainly lactic acid bacteria (LAB), coagulase-negative staphylococci (CNS), yeasts, and molds. LAB converts fermentable sugars to lactic acid by creating unfavorable conditions for pathogens (such as *Escherichia coli*, *Listeria monocytogenes,* and *Yersinia enterocolitica*) and/or spoilage microorganisms—in fact, a rapid acidification is important for safety, while a high competitiveness against the spontaneous lactic flora is important for product quality [2]. On the other hand, the correct fermentation of meat products could counteract the waste of spoiled products—nowadays, food waste is a challenge worldwide [3], and the use of starter cultures with a strong competitive potential towards spoilers and good technological properties (acidification and survival within the storage) could assure a longer shelf life and reduce waste.

The most frequent LAB species recovered in meat are *Lactobacillus sakei*, *Lb. curvatus*, *Lb. plantarum*, and *Lb. casei*. However, the contribution of enterococci also seems to be relevant [4].

Processed meat foods do not undergo heat treatment—thus, they could be suitable carriers for functional starter cultures; that is, beneficial microorganisms acting at the same time as starter cultures and probiotics [5]. Many researchers have addressed this topic—for example, Coelho et al. [6] and Ge et al. [7] applied *Lb. paracasei* LPC02 and *Lb. plantarum* NJAU-01 in the processing of fermented sausages with improvements in their technological characteristics. Macedo et al. [8] and Rebucci et al. [9] suggested the use of strains of *Lb. casei*, *Lb. paracasei*, and *Lb. rhamnosus* as potential functional starter cultures in meat products. However, these microorganisms could suffer from the potential negative impact of the meat environment—in particular its high content in curing salt and its low pH and water activity.

A solution to overcome this challenge is the use of autochthonous LAB, because they are desirable for: (i) their ability to adapt to the ecological conditions of specific meat fermentations, (ii) control of the ripening processes, and (iii) the ability to inhibit the growth of spontaneous microorganisms—therefore autochthonous starters are recommended to achieve the desired fermentation parameters specific for the product type [3]. In a previous project [10,11], the autochthonous LAB of meat were assessed for their functional properties—as a result, *Lb. plantarum* 178 was selected as a promising functional starter culture because of its technological performance (acidification, growth in presence of salt, good interaction with staphylococci CNS), and functional traits (hydrophobicity, survival to simulated gastro-intestinal conditions, antimicrobial activity).

The aim of this paper is to propose an industrial validation of *Lb. plantarum* 178 to improve the quality of Italian sausages. The performances of this strain were preliminarily compared to a commercial probiotic (*Lb. casei* LC01) and to a commercial starter (*Lb. sakei* ST2), and then validated in an industrial process.

## 2. Materials and Methods

### 2.1. Microorganisms

Three strains were used in this research: (i) *Lb. plantarum* 178, isolated from pork meat, a functional starter with probiotic characteristics [11]; (ii) *Lb. casei* LC01, Chr. Hansen (Hørsholm, Denmark); and (iii) *Lb. sakei* ST2, isolated from a commercial preparation [1]. The strains were stored at −20 °C in MRS broth (Oxoid, Milan, Italy), and were added with 33% (mL·L^−1^) of sterile glycerol (J.T. Baker, Milan, Italy). Before each assay, the microorganisms grown in MRS broth were incubated at 30 °C for 24–48 h.

### 2.2. Acidification

Acidification (pH decrease, ΔpH) was assessed in a meat model system (beef extract, 5 g·L^−1^; bacteriological peptone, 10 g·L^−1^; tryptone, 5 g·L^−1^; NaNO_2_, 150 mg·L^−1^; NaNO_3_, 250 mg·L^−1^; NaCl, 50 g·L^−1^; lactose, 4 g·L^−1^; black pepper, 1 g·L^−1^), inoculated to 7 log cfu·mL^−1^ and incubated at 15, 20, 25, 30 and 44 °C. The pH was measured after 6 and 24 h through a pH meter Crison 2001 (Crison Instruments, Barcelona, Spain).

### 2.3. Bioactivity toward Foodborne Pathogens

The interaction of *Lb. plantarum* 178, *Lb. casei* LC01 and *Lb. sakei* ST2 with *Staphylococcus aureus*, *Listeria monocytogenes*, *Escherichia coli*, *Salmonella* sp. was screened by an agar well diffusion method. The pathogens belong to the Culture Collection of the Department of the Science of Agriculture, Food and Environment, University of Foggia—before each experiment, the pathogens were grown in Nutrient Broth (Oxoid) incubated at 37 °C for 24 h.

Pathogens were separately inoculated to 7 log cfu·mL^−1^ on nutrient agar plates, then wells of 9 mm of diameter were cut off through a cork borer and 80 µL of the following solution was distributed: (i) Solution A: LAB culture in MRS broth (ca. 7 log cfu·mL^−1^); (ii) Solution B: not-buffered LAB supernatant (cell-free) prepared by centrifuging cell culture at 1000× *g* for 10 min; and (iii) Solution C: cell-free LAB supernatant buffered to pH 6.0 through NaOH 1 mol L^−1^. Distilled water was used as a negative control.

The tests were performed on three independent batches. The plates were incubated at 15, 25 and 30 °C for 24–48 h. An inhibition zone around the well was assumed to be a significant antimicrobial action of LAB against the pathogens.

### 2.4. Industrial Fermentation

The experiments were performed as follows: (i) sausages inoculated with *Lb. plantarum* 178; (ii) sausages inoculated with *Lb. casei* LC01; and (iii) uninoculated sausages.

The strains had been previously freeze-dried, and the viable count was ca. 10 log cfu·g^−1^. The mixture was prepared according to the traditional formulation of sweet Calabrian salami—lean pork meat (69.5%), lard (17.5%), NaCl (2.2%), dextrose (0.4%), sodium ascorbate E301 (0.2%), fennel seeds (0.2%), sweet Eurodroga Calabra (Europrodotti SpA, 0.5%), bell pepper extract (8.6%), and freeze-dried strains (0.9%). The mixture (300 kg) was then stuffed into a natural casing—the weight of each sausage was 300–400 g. Sausages were ripened in an industrial plant as follows: stewing stage (4 h at 22 °C, relative humidity (RH) 99%); drying stage (7 h at 22 °C, RH 65%); intermediate drying/ripening stage (4 days, with the temperature decreasing from 20 °C to 15 °C, and RH increasing from 67% to 73%); first ripening stage (5 days at 15 °C, RH 71%); second ripening stage (5 days at 13 °C, RH 73%), and a final ripening/maturation stage (15 days at 12 °C, RH 75%).

For microbiological analysis, the casing was discarded and 25 g of the inner part was randomly selected, diluted with sterile saline solution (0.9% NaCl) (1:10), and homogenized using a Stomacher LAB Blender 400 (Pbi International, Milan, Italy). The homogenates were serially diluted and plated on selective media and incubated under appropriate conditions.

The media and the conditions used were: Plate Count Agar (PCA, Oxoid, Milan, Italy) incubated at 30 °C for 24–48 h for mesophilic bacteria; Violet Red Bile Glucose Agar (VRBGA) incubated at 37 °C for 18–24 h for Enterobacteriaceae; MRS agar + cycloheximide (0.17g·L^−1^) (Sigma-Aldrich, Milan, Italy) incubated at 30 °C for 72 h under anaerobiosis for LAB; Sabouraud Dextrose Agar + chloramphenicol (0.1 g·L^−1^) (Carlo Erba, Milan, Italy) incubated at 28 °C for 48–72 h for yeasts; Malt Extract Agar (MEA) incubated at 25 °C for 4 days for molds; Pseudomonas Agar Base + CFC supplement for Pseudomonadaceae at 25 °C for 48–72 h; Mannitol Salt Agar for Staphylococci and micrococci at 37 °C for 24–48 h; Baird-Parker Agar Base for coagulase-positive staphylococci at 37 °C for 24–48 h (coagulase-positive staphylococci were evidenced by a clear halo around the colonies); and SPS Agar for sulphite-reducing Clostridia at 37 °C for 24 h under narrow anaerobic conditions. All media used were from Oxoid (Milan, Italy). In addition, MacConkey MUG Agar for *E. coli* at 37 °C for 24–48 h, X.L.T. Agar for *Salmonella* sp. for 24–48 h, and Listeria Palcam Agar for *L. monocytogenes* at 37 °C for 24–48 h were used. All media used for these pathogens were from Liofilchem (Roseto degli Abruzzi, Italy). The results for plate counts were confirmed by microscopic observations.

The following physico-chemical analyses were done:

(a) The pH was measured on sausage homogenate through a pH meter Crison 2001 (Crison Instruments, Barcelona, Spain).
(b) Color was monitored by colorimetric measurements using a Tristimulus Colorimeter Chromameter-2 Reflectance (Minolta, Osaka, Japan), equipped with a CR-300 measuring head. The instrument was standardized against a white tile before each determination. The color of the sausages was determined by a Hunter scale as L * (brightness), a * and b * (hue and saturation of the color). Data were the average of at least five repetitions.
(c) Water activity measurements were performed by using a hygrometer AQUA LAB CX-2 (Decagon Device, Pullman, WA, USA).
(d) Moisture content was measured by using Sartorius Thermal Balance (Antela, Florence, Italy), at 130 °C until the samples reached a constant weight.

### 2.5. Statistic

The experiments were performed on at least two independent batches (three batches for the antimicrobial activity assay)—for each batch, the analyses were made twice. Significant differences were pointed out through one-way analysis of variance or two-way analysis of variance (ANOVA) and Tukey’s test, as the post-hoc comparison test (*p* < 0.05). Statistics was made through the software Statistica for Windows (Statsoft, Tulsa, OK, USA).

## 3. Results and Discussion

### 3.1. Preliminary Validation

Fermented sausages are part of Italian tradition, but they are often the result of spontaneous uncontrolled fermentations, therefore several drawbacks can occur, such as biogenic amine production or the growth of unsuitable microorganisms [12]. Since many technological properties of food-grade microorganisms are strain-specific, the selection of an autochthonous strain could give safe, predictable, and constant results [13,14].

The main bacteria having an important role in fermented sausages are lactic acid bacteria (LAB), as they are used as starter cultures to promote meat fermentation [1]. However, the biodiversity of commercial starter cultures is limited. Therefore, the selection and development of new starter cultures from the native microbiota of sausages produced by spontaneous fermentation, as well as their use in the production of sausages, can result in the development of products with a high level of hygiene and regional products with specific sensory characteristics [15,16]. Wild LAB are well-adapted to ecological niches and are a good reservoir of promising functional and starter strains [4,17].

Carnevali et al. [18] proposed a general scheme for the selection of a starter: first, a lab phase (isolation, technological characterization, and selection of interesting strains); a validation on a lab scale, and a third phase focusing on the use of the starter at the industrial level (scale up).

This research focuses on the third step (industrial validation) for the autochthonous strain *Lb. plantarum* 178, with some confirmatory experiments done at lab level (acidification and antimicrobial activity)— the technological and functional characterization is proposed elsewhere [11].

**Figure 1:**
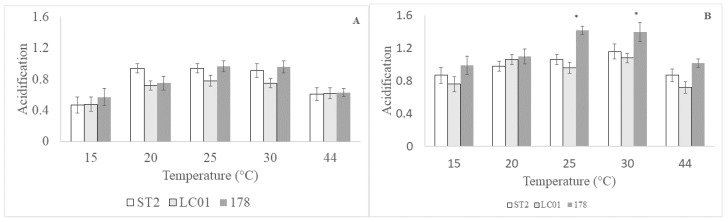
Acidification (ΔpH) performed by *Lb. sakei* ST2, *Lb. casei* LC01 and *Lb. plantarum* 178 in a synthetic medium simulating meat after 6 (**A**) and 48 h (**B**). Mean ± standard deviation. The symbol “*” indicates a significant difference (two-way ANOVA and Tukey’s test, *p* < 0.05).

Figure 1 shows acidification of a synthetic medium by the three targets (*Lb. plantarum* 178, *Lb. sakei* ST2, and *Lb. casei* LC01) after 6 h (Figure 1A) and 24 h (Figure 1B) at different temperatures (15, 20, 25, 30 and 44 °C). After 6 h (Figure 1A), ΔpH was not affected by the strains, but it was significantly influenced by the temperature (Table 1). In fact, the strains experienced the highest acidification scores at 25 and 30 °C (0.78 and 0.97)—at the lowest and the highest temperature (15 and 44 °C) acidification was 0.47 and 0.63, respectively, without significant differences.

After 24 h, acidification was affected by the interaction temperature/strains, while neither temperature nor strains were a significant factor. At 25–30 °C, *Lb. plantarum* 178 experienced a higher ΔpH (1.4 for *Lb. plantarum* 178 and 1.0–1.1 for *Lb. casei* LC01 and *Lb. sakei* ST2, respectively) (Figure 1B). In addition, the strains were able to perform acidification at both 15 and 44 °C without significant differences (ΔpH, 0.72–1.02).

Acidification is a desirable technological characteristic, since it is a simple index with which to evaluate the performance of a starter. The production of organic acids during fermentation allows the reaching of a very low pH, preventing the growth of pathogens during ripening [19]. Moreover, lactic acid, as well as other weak acids, has positive effects on the flavor because, when combined with ethanol and other products, it strengthens the perception of aroma [14].

The acidification, at 15 °C as well as at 44 °C, of *Lb. plantarum* 178 suggests that it is a robust microorganism able to start fermentation at low temperatures (15–18 °C) and perform an acidification kinetic at high temperatures (44 °C). This is an important trait because temperature is the limiting factor for the selection of starter cultures for sausages [13]. Moreover, *Lb. plantarum* 178 experienced acidification in a meat-simulating medium containing the highest concentrations of nitrates and nitrites allowed by current legislation and a concentration of NaCl 5% higher than the typical amount used in Italian sausages (2.5–4%) [20,21].

Another important characteristic to select a functional starter is the bioactivity toward pathogens. As suggested by Babic et al. [22], *S. aureus*, *L. monocytogenes*, *E. coli*, and *Salmonella* sp. were investigated. Table 2 shows the results for the *Lb. plantarum* 178 strain, since *Lb. casei* and *Lb. sakei* do not produce an inhibition halo. *Lb. plantarum* cultures always inhibited *E. coli*, *Salmonella* sp., and *S. aureus*, while *L. monocytogenes* was inhibited at 25 and 30 °C, but not at 15 °C. Similar trends were found with the supernatant, but not with the buffered supernatant. Babic et al. [22] reported that pathogens could be inhibited by a combined action of low pH competition for substrates and/or bacteriocin production—however, the results of the antimicrobial assay suggest that the bioactivity of *Lb. plantarum* 178 towards pathogens was mainly due to acidification.

### 3.2. Validation at Industrial Level

As a final step, *Lb. plantarum* 178 was validated at an industrial level and compared to a functional starter culture with similar traits (LC01). Uninoculated sausages experienced a slight decrease of pH from 5.6 to 5.3—lab counts were at 3–4 log cfu·g^−1^ for the whole fermentation, and Enterobacteriaceae attained a cell count of 7.3 log cfu·g^−1^ after 10 days. Humidity did not decrease (35–37%) and activity water (a_w_) was always at 0.95 (data not shown).

**Figure 2:**
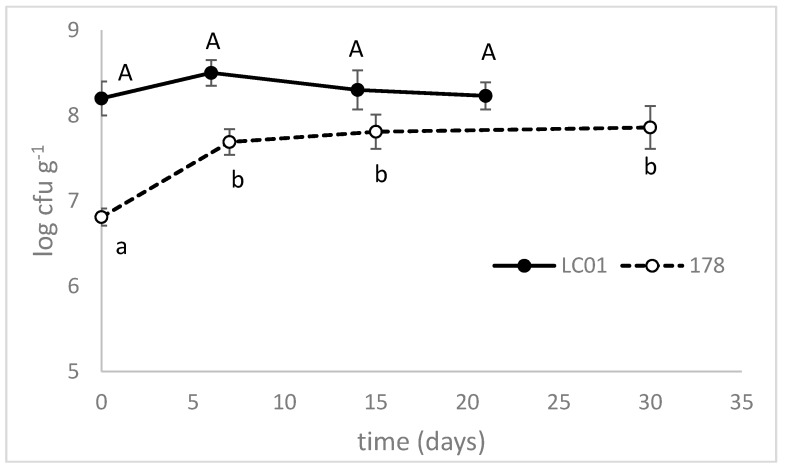
Lactic acid bacteria (LAB) counts, during the ripening of traditional sausages inoculated with *Lb. casei* LC01 or *Lb. plantarum* 178. Mean ± standard deviation. The letters indicate statistically significant differences throughout time (one-way ANOVA and Tukey’s test, *p* < 0.05): small letters, sausages inoculated with *Lb. plantarum* 178; capital letters, LC01.

Figure 2 shows lactic acid bacteria counts in inoculated sausages—because of the initial inoculum in LC01-sausages, the LAB count was 8.2 log cfu·g^−1^, which did not undergo significant changes during fermentation and ripening (21 days). In 178-inoculated sausages, the LAB count experienced a slight increase (from 7 to 8 log cfu·g^−1^).

**Figure 3:**
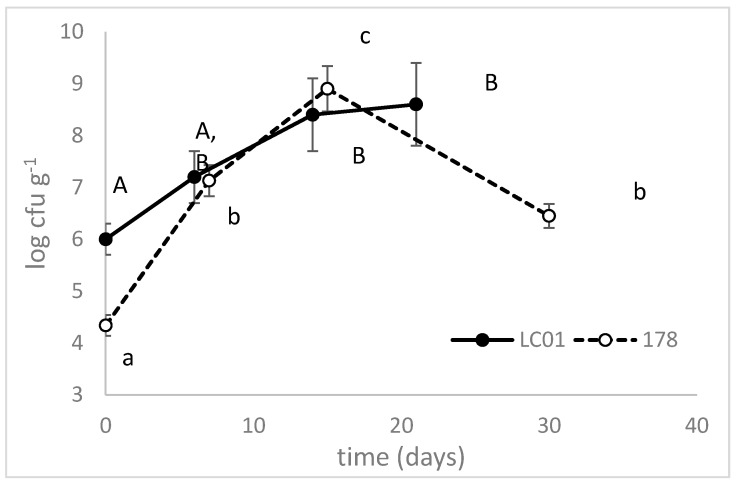
Micro-staphyoloccci counts during the ripening of traditional sausages inoculated with *Lb. casei* LC01 or *Lb. plantarum* 178. Mean ± standard deviation. The letters indicate statistically significant differences throughout time (one-way ANOVA and Tukey’s test, *p* < 0.05): small letters, sausages inoculated with *Lb. plantarum* 178; capital letters, LC01.

In LC01-sausages, micrococci and staphylococci increased from 6 log cfu·g^−1^, immediately after mixing and sausage preparation, to 8.5 log cfu·g^−1^ after 14 days (Figure 3). In 178-inoculated sausages, micro-staphylococci increased from 4.34 to 8.9 log cfu·g^−1^ after 14 days, then experienced a decrease with a final count of 6.45 log cfu·g^−1^.

Staphylocci play a major role in sausage fermentation and they are often used as starter cultures in combination with LAB because they consume oxygen and possess nitrate/nitrite reductase and catalase activities. In addition, their specific protease and peptidase activity can affect the flavor profile in sausages. In some Italian traditional sausages (like Felino salami), they are used as starter cultures without the addition of LAB because their activity imparts to the sausage a “sweet” rather than a “sour” taste, which is preferred by consumers [23]. However, this practice is a bad way to conduct fermentation because it does not assure a correct acidification kinetic [24].

**Figure 4:**
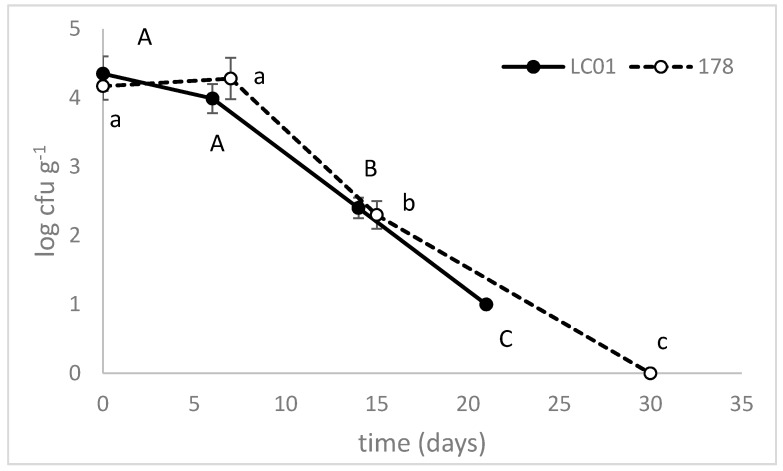
Enterobacteria counts, during the ripening of traditional sausages inoculated with *Lb. casei* LC01 or *Lb. plantarum* 178. Mean ± standard deviation. The letters indicate statistically significant differences throughout time (one-way ANOVA and Tukey’s test, *p* < 0.05): small letters, sausages inoculated with *Lb. plantarum* 178; capital letters, LC01.

Due to lactic fermentation, Enterobacteriaceae (4–5 log cfu·g^−1^ at the beginning) decreased throughout ripening (Figure 4); *Salmonella* sp., *E. coli*, *L. monocytogenes* and Clostridia were always below the detection limit (data not shown).

**Figure 5:**
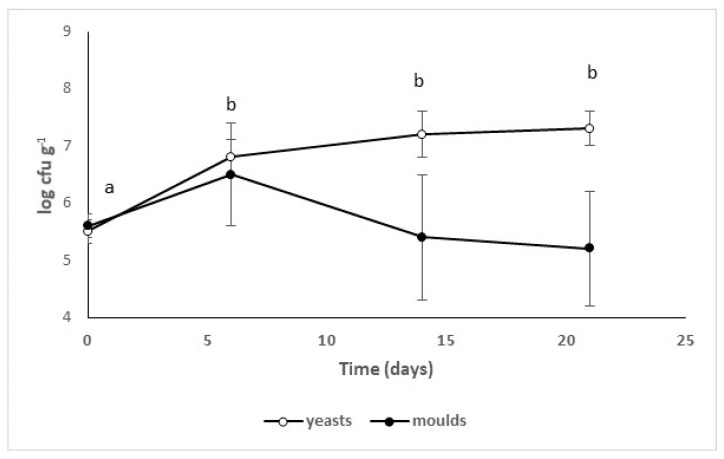
Yeasts and molds during the ripening of traditional sausages inoculated with *Lb. casei* LC01. Mean value ± standard deviation. The letters and the symbols indicate significant differences for each microbial group (one-way ANOVA and Tukey’s test, *p* < 0.05).

Yeasts and molds played a key role during the last stages of ripening. Figure 5 shows yeasts and molds in sausages inoculated with *Lb. casei* LC01—yeasts increased from 5.5 log cfu·g^−1^ (immediately after bagging) to ca. 7.5 log cfu·g^−1^ after 14 days. On the other hand, the trend of molds was extremely variable, and a macroscopic examination suggested the predominance of *Penicillium* spp.

The evolution of fermentation and ripening was evaluated through color, pH, a_w_, and humidity—concerning color, hue angle values were always in the range of purple (data not shown).

**Figure 6:**
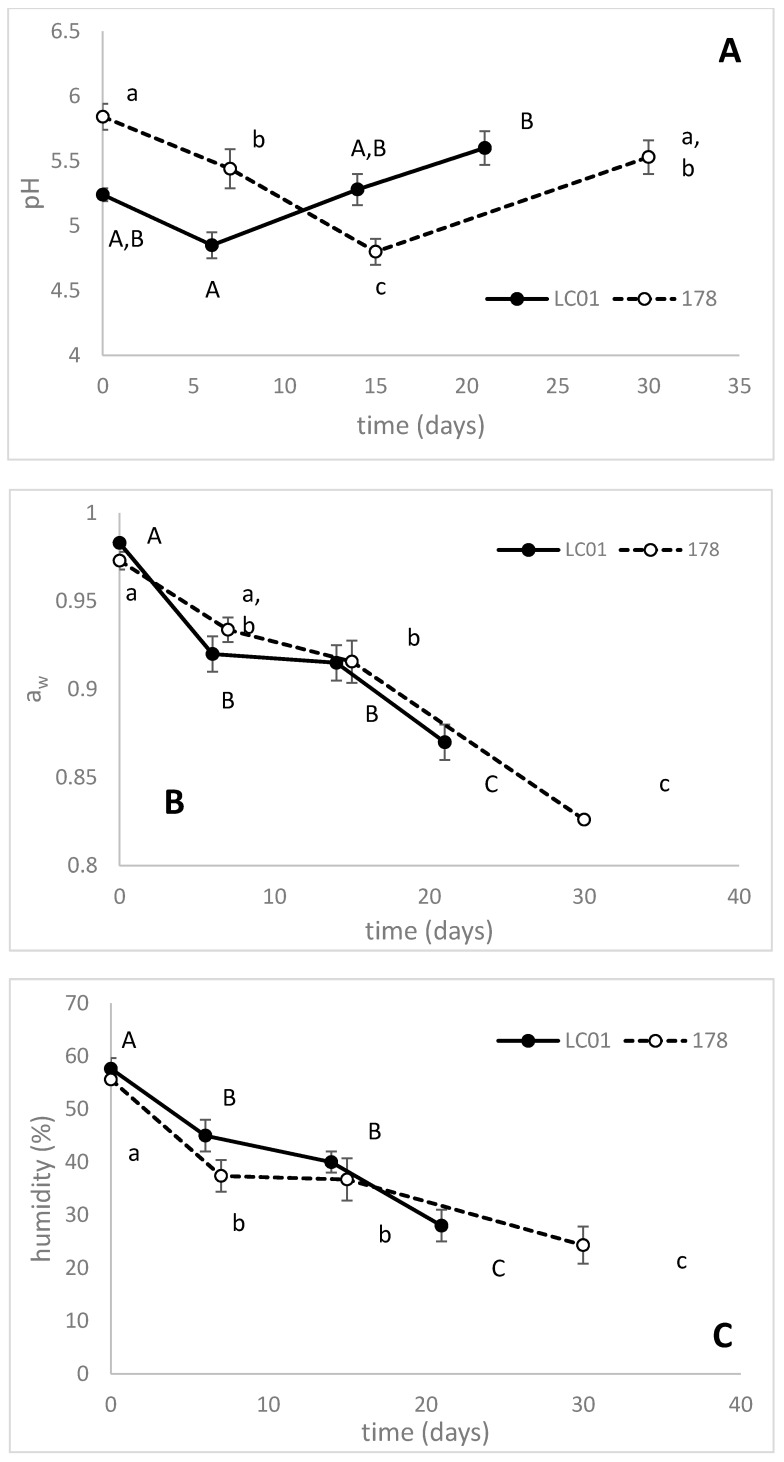
pH (**A**), a_w_ (**B**) and relative humidity (**C**) in traditional sausages inoculated with *Lb. casei* LC01 or *Lb. plantarum* 178. Mean ± standard deviation. The letters indicate statistically significant differences throughout time (one-way ANOVA and Tukey’s test, *p* < 0.05): small letters, sausages inoculated with *Lb. plantarum* 178; capital letters, LC01.

Figure 6 shows pH, humidity, and a_w_. The pH of both LC01 and 178-sausages showed the typical trend of sausage fermentation (salame nostrano and soppressata molisana) [20,21,23] with an initial decrease, followed by an increase to 5.5–5.6 (Figure 6A)—a_w_ also followed a typical trend, with a final value of 0.82 for 178-sausages and 0.87 for LC01-sausages at the end of fermentation (Figure 6B). In general, the final pH of the fermented sausages in several countries was highly variable, with an average value of approximately 5.0 [25,26,27,28,29].

a_w_ reduction, along with acidification, is a desirable trait—depending on the temperature, pathogen inhibition could be either the results of pH or a_w_ [19]. When the temperature is lower than 20 °C, a_w_ reduction is more important than a decrease in pH [30]. At higher fermentation temperatures (>20 °C), the crucial factor for *L. monocytogenes* and *Salmonella* sp. inhibition is acidification [30].

The pH reduction favored the irreversible denaturation of proteins and the correct release of water and the cohesion of the dough during ripening—in fact, the humidity decreased from 55% to 28% (LC01), or 24% in the case of *Lb. plantarum* 178 (Figure 6C). These events favored a correct inhibitory and selective action on the microorganisms of the mixture and assured the safety of the product. In fact, the microbial stability of sausages can be assured by the combination of low pH (4.6–5.9) and water activity (<0.9) [31], therefore the *Lb. plantarum* 178 strain can be considered to be an optimal strain, able to start, conduct, and complete fermentation with similar performances compared to LC01. Moreover, the performance of *Lb. plantarum* 178 was similar to some other autochthonous cultures used in other traditional products in terms of acidification, reduction of a_w_ and humidity [20,23], and microbiota imprinting with pathogens below the detection limit and spoilers at low amounts.

## 4. Conclusions

The choice of an appropriate microorganism to improve the performance of a fermented meat matrix is very important—for an autochthonous strain, the transition from a laboratory to an industrial scale is a complex process, since the repeatability of the results is not guaranteed and many times strains that are promising at lab level cannot control an industrial process. This study represents the scaling up and the validation of a promising autochthonous strain (*Lb. plantarum* 178) at an industrial level and shows the possibility of performing the fermentation of traditional Italian sausage through functional starter cultures, combining the benefit of a controlled fermentation and possible health benefits due to the functional properties of the strain. From a practical point of view, *Lb. plantarum* showed some interesting technological properties: (i) a good acidification score at 25–30 °C, higher than the value found for two commercial strains; (ii) the ability to perform acidification at 15 and 44 °C; (iii) the ability to perform fermentation under real conditions, thus assuring a correct course for the physico-chemical parameters (a_w_, pH, humidity); and good microbiological imprinting.

## Figures and Tables

**Table 1:** Two-way analysis of variance performed on the acidification scores after 6 and 24 h—ns, not significant; * *p* < 0.05; ** *p* < 0.01.

	6 h	24 h
Temperature	**	ns
Strains	ns	ns
Temperature/strains	ns	*

**Table 2:** Inhibition halo ([Halo]–[well diameter]) of *Lb. plantarum* 178 towards pathogens. The data are the average of three repetitions. -, no halo.

		15 °C	25 °C	30 °C
**Cell Culture**	*E. coli*	0.55	0.5	0.45
	*L. monocytogenes*	-	1	1.4
	*Salmonella*	0.8	0.35	0.3
	*Staph. aureus*	0.95	0.7	0.9
**Supernatant**	*E. coli*	0.45	0.35	0.3
	*L. monocytogenes*	0.5	0.25	0.4
	*Salmonella*	0.55	0.35	0.3
	*Staph. aureus*	0.5	0.4	0.3
**Buffered Supernatant**	*E. coli*	-	-	-
	*L. monocytogenes*	-	-	-
	*Salmonella*	-	-	-
	*Staph. aureus*	-	-	-
